# A randomized phase II study of aromatase inhibitors plus metformin in pre-treated postmenopausal patients with hormone receptor positive metastatic breast cancer

**DOI:** 10.18632/oncotarget.20478

**Published:** 2017-08-24

**Authors:** Yannan Zhao, Chengcheng Gong, Zhonghua Wang, Jian Zhang, Leiping Wang, Sheng Zhang, Jun Cao, Zhonghua Tao, Ting Li, Biyun Wang, Xichun Hu

**Affiliations:** ^1^ Department of Medical Oncology, Fudan University Shanghai Cancer Center, and Department of Oncology, Shanghai Medical College, Fudan University, Shanghai 200032, China

**Keywords:** metastatic breast cancer, hormone receptor positive, endocrine therapy, metformin, pre-treated

## Abstract

**Background:**

Everolimus significantly improves progression-free survival (PFS) and has been approved to use in aromatase inhibitor pretreated patients with hormone receptor positive advanced breast cancer. Metformin has been shown to inhibit mTOR pathway, with more favorable safety profile, leading to this hypothesis-generating trial to assess whether metformin enhances the efficacy of aromatase inhibitors.

**Methods:**

60 postmenopausal women with hormone receptor positive locally advanced or metastatic breast cancer were randomly assigned 1:1 to aromatase inhibitor (exemestane 25mg/d or letrozole 2.5mg/d depending on the most recent treatment) plus metformin (0.5g bid, orally) or placebo. The primary endpoint was PFS, and secondary endpoints were objective response rate, clinical benefit rate, overall survival and safety.

**Results:**

Median PFS was 4.7 months in the combination group and 6.0 months in the control group (hazard ratio, 1.2; 95% confidence interval [CI], 0.7 to 2.1; P =0.48). ORR was 6.7% in the combination group and 0% in the control group (odds ratio for ORR not available; P =0.99), and CBR was 33.3% and 50.0%, respectively (OR for CBR 0.5; 95% CI, 0.2 to 1.4; P=0.15). No significant difference in overall survival was observed between the combination and control groups (median OS, 30.9 vs. 32.4 months; P = 0.81). Subgroup analyses didn't find any specific population favoring the combination treatment. No substantial difference in incidence or severity of adverse events was seen between the two treatment groups.

**Conclusion:**

This randomized phase II clinical trial failed to show an improved efficacy with the addition of metformin to endocrine therapy, although with excellent tolerability.

## INTRODUCTION

The biguanide metformin is one of the backbone drugs in the treatment of hyperglycemia and type 2 diabetes [1]. It exerts its hypoglycemic action by sensitizing peripheral tissues to insulin, increasing insulin-dependent glucose uptake of cells, inhibiting gluconeogenesis, and decreasing glucose absorption in the small intestine [2].

Recently, metformin has being assessed as an anti-cancer agent [3, 4]. Retrospective epidemiological studies based on public databases, comprehensive literature search and meta-analysis compared tumor incidence and cancer-related mortality of diabetic patients taking metformin with those taking other hypoglycemic agents as well as non-diabetic patients. It was concluded that tumor incidence and cancer-related mortality decreased with metformin treatment [5–9]. Possible molecular mechanisms include direct (insulin-independent) and indirect (insulin-dependent) actions of the drug [10]. The direct effects of metformin mainly occur through the induction of AMP-activated protein kinase (AMPK), consequently reducing mammalian target of rapamycin (mTOR) signaling and protein synthesis in cancer cells [11]. The indirect effects are the downstream response to AMPK activation. Metformin inhibits the transcription of key gluconeogenesis genes in the liver and reduces blood glucose and insulin [2]. Thus, it inhibits insulin-induced proliferation of tumor cells [12, 13].

Several clinical studies have confirmed the antitumor effects of metformin in breast cancer patients, especially those with diabetes or other metabolism disorders. A retrospective clinical study showed that patients treated with metformin during neoadjuvant chemotherapy have a higher pathologic complete response (pCR) rate than diabetic and non-diabetes patients not administered metformin [14]. Furthermore, a Chinese phase II randomized clinical trial demonstrated an increased pCR rate in early breast cancer patients after addition of metformin (0.25g tid) to routine chemotherapy regimens (cyclophosphamide + epirubicin + 5-fluorouracil) [15]. In addition to efficacy outcomes, several surrogate markers of relevant signaling pathways have been described in breast cancer. These include Ki67, S6K, 4E-BP-1, AMPK and other key molecules of the AMPK/mTOR pathway [16]. Overall, most studies indicated reduced ki67 and increased TUNEL levels after metformin administration [16, 17].

Emerging evidence demonstrates that PI3K/Akt/mTOR activation is an important mechanism of acquired endocrine resistance [18]. Everolimus, an inhibitor of mTOR, reverses resistance to endocrine therapy, and is effective in patients who experience progression after prior endocrine treatments [19, 20]. BOLERO-2 demonstrated that everolimus combined with exemestane (steroidal AI) improves progression-free survival (PFS) in advanced breast cancer patients previously treated with endocrine therapies, compared with the exemestane monotherapy group [19].

Metformin inhibits the activation of mTOR and relevant proliferation signaling pathways. Since everolimus was not approved by CFDA and unavailable in China at the time of study design, we assessed whether metformin could constitute a substitute for everolimus in the treatment of patients with hormone receptor (HR) positive breast cancer. Thus, we hypothesized that metformin could further enhance the efficacy of aromatase inhibitors, in patients with HR-positive advanced breast cancer.

## RESULTS

### Patients

**Figure 1:**
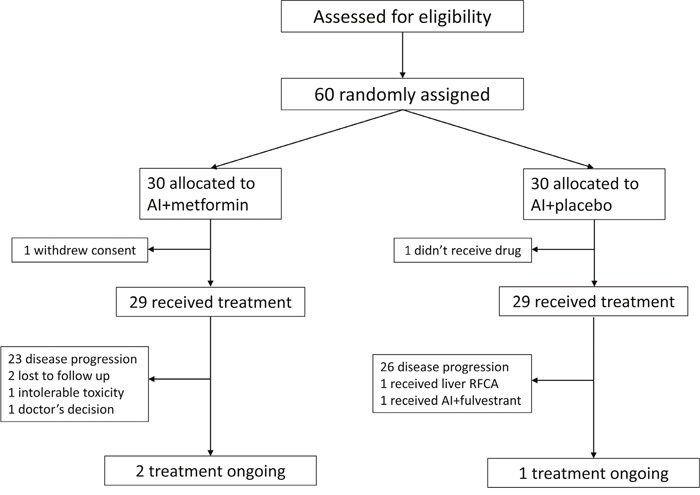
Consort graph

In total, 60 patients were randomly assigned, 30 each to the AI plus metformin and AI groups (Figure 1). The eligible patients were enrolled between Jun 17, 2012 and Oct 14, 2014. Baseline characteristics, including treatment history, were well balanced across the treatment groups (Table 1). Median age was 57 years (range, 33-73). A total of 13 (43.3%) patients had visceral metastases in the combination group, and 18 (60.0%) in the control group. 14 (46.7%) and 8 (26.7%) patients had first-line endocrine therapy in the combination and control groups, respectively. The patients were divided by sensitivity to previous endocrine therapy (Table 1). Most patients in both groups showed secondary resistance. In addition, the most recent endocrine therapy included antiestrogen agents (16.7% and 33.3% in the combination and control groups, respectively) and AI (83.3% and 66.7% in the combination and control groups, respectively).

**Table 1:** Patient characteristics

Characteristic	AI +Met (*n* = 30)No.(%)	AI (*n* = 30)No.(%)
Median age, years (range)	57.5(33-72)	56.5(33-73)
De novo stage IV	1(3.3)	2(6.7)
Disease free interval^*^		
Median(year)	5.4	5.7
Range(year) ≤24 mo-No.(%) >24 mo-No.(%)	1.8-14.92(6.7)27(90.0)	0.5-14.05(16.7)23(76.7)
Measurable lesion-No.(%)	19(63.3)	16(53.3)
No. of metastatic sites-No.(%)		
1	12(40.0)	11(36.7)
2	9(30.0)	12(40.0)
≥3	9(30.0)	7(23.3)
Metastatic sites-No.(%)		
Visceral	13(43.3)	18(60.0)
Lung	10(33.3)	14(46.7)
Liver	5(16.7)	3(10.0)
Non-visceral	17(56.7)	12(40.0)
Bone alone	5(16.7)	6(20.0)
Most recent endocrine therapy-No.(%)		
Adjuvant endocrine therapy	14(46.7)	8(26.7)
Endocrine therapy for MBC	16(53.3)	22(73.3)
Lines of endocrine therapy for MBC-No.(%)		
First line	14(46.7)	8(26.7)
Second line	15(50.0)	20(66.7)
Third or more line	1(3.3)	2(6.7)
Sensitivity to previous endocrine therapy#-No.(%)		
Primary resistance	3(10.0)	5(16.7)
Secondary resistance	24(80.0)	21(70.0)
Naïve	2(6.7)	2(6.7)
Not evaluable	1(3.3)	2(6.7)
Most recent treatment-No.(%)		
Antiestrogen	5(16.7)	10(33.3)
AI	25(83.3)	20(66.7)
Previous lines of chemotherapy for MBC-No.(%)		
0	20(66.7)	22(73.3)
1 ≥2	4(13.3)6(20.0)	7(23.3)1(3.3)

^*^Disease-free interval is defined as the time from diagnosis of breast cancer to first relapse (29 patients in the combination group and 28 patients in single-agent group).

# Primary endocrine resistance is defined as: a relapse while on the first 2 years of adjuvant endocrine therapy, or PD within first 6 months of first-line endocrine therapy for MBC, while on endocrine therapy. Secondary (acquired) endocrine resistance is defined as: a relapse while on adjuvant endocrine therapy but after the first 2 years, or a relapse within 12 months of completing adjuvant endocrine therapy, or PD ≥6 months after initiating endocrine therapy for MBC, while on endocrine therapy.

### Efficacy

**Figure 2:**
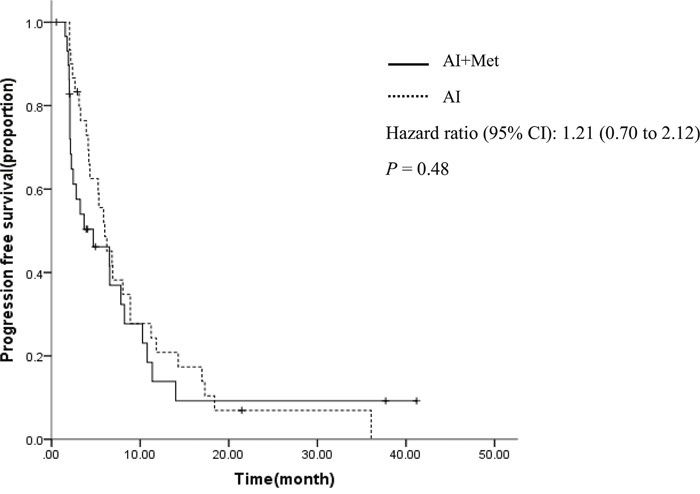
Progression-free survival curves by treatment arm

The cutoff date for the study was Jul 10, 2016. All randomized patients were included in this analysis (Figure 1). Efficacy evaluations were listed in Table 2. Treatment with AI plus metformin, compared with placebo plus AI, did not significantly improve PFS. Treatment in the combination group resulted in a median PFS of 4.7 months, and 6.0 months in the control group (hazard ratio [HR], 1.2; 95% CI, 0.7 to 2.1; P =0.48; Figure 2), which is based on intention-to-treat analysis; 49 progression events were obtained, with 23 (76.7%) in the combination group and 26 (86.7%) in the control group.

**Table 2:** Evaluation of efficacy

Variable	AI +Met (*n* = 30)No.(%)	AI (*n* = 30)No.(%)
Progression-free survival		
Events — No. (%)	23(76.7)	26(86.7)
Duration — mo		
Median	4.7	6.0
95%CI	0.3-9.0	4.0-7.9
Overall survival		
Events — No. (%)	13(25.0)	12(39.3)
Duration — mo		
Median	30.9	32.4
95%CI	NR	23.8-41.0
Best overall response-No.%		
Complete response	0(0.0)	0(0.0)
Partial response	2(6.7)	0(0.0)
Stable disease	14(46.7)	22(73.3)
Duration of SD ≥24 weeks	8(26.7)	15(50.0)
Progression disease	12(40.0)	7(23.3)
NE	2(6.7)	1(3.3)
ORR	2(6.7)	0
CBR	10(33.3)	15(50.0)

**Figure 3:**
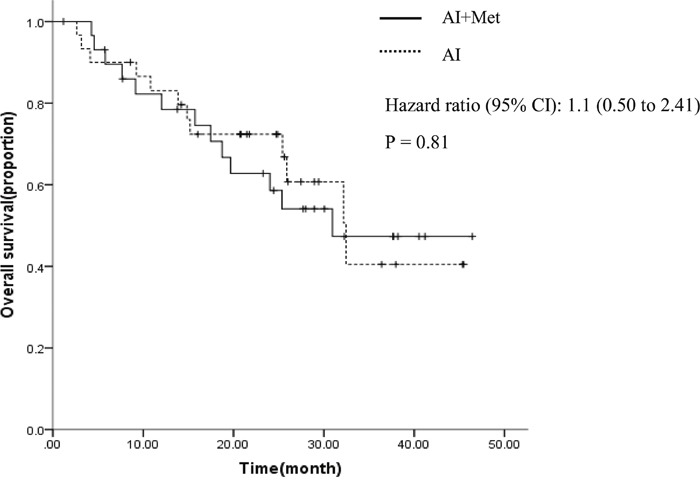
Overall survival curves by treatment arm

ORR was 6.7% (95%CI 0.3-16) in the combination group, and 0% (95%CI not available) in the control group. CBR was 33.3% (95%CI 15-51) in the combination group, and 50.0% (95%CI 31-69) in the control group. AI and metformin combination was not associated with increased ORR (OR not available P=0.99) and CBR (OR=0.50, 95% CI 0.2 to 1.4, P=0.15). Median follow-up was 22.3 months. A total of 13 (43.3%) patients died in the combination group, and 12 (40.0%) in the control group. Median OS times were 30.9 and 32.4 months for the combination and control groups, respectively (HR= 1.1, 95%CI 0.5 to 2.4, P = 0.81, Figure 3).

**Figure 4:**
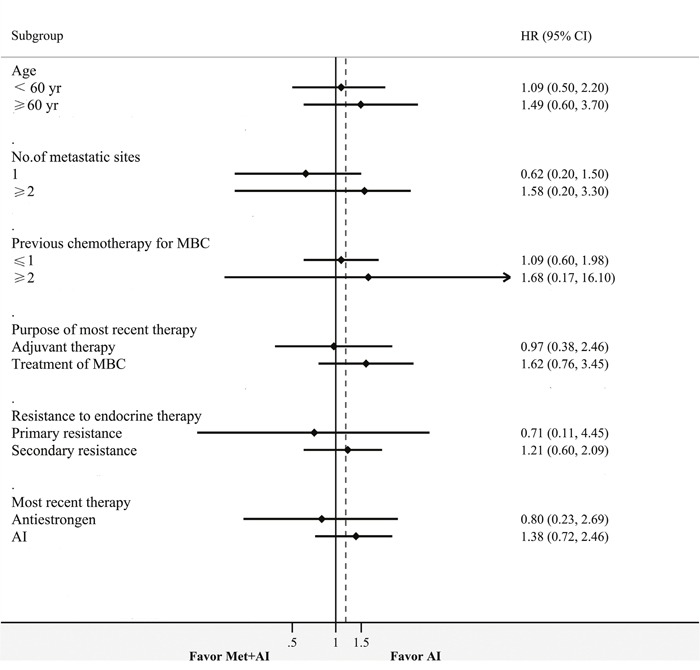
Forest plot of PFS

In subgroup analysis, treatment effects were consistent across all predefined subgroups. A numerical PFS increase was obtained in the combination group in patients with primary resistance compared to those with secondary resistance (10.8 vs. 5.8 months, p=0.71). Patients had slightly but non-statistically signifcant PFS increase after last antiestrogen therapy in the combination group compared with the control group (10.8 vs. 4.3 months, p=0.72) (Table 3, Figure 4). Patients with primary resistance benefited from metformin, with risk of progression numerically reduced by 29% (HR=0.7, 95% CI 0.1 to 4.4; Table 3 and Figure 4). A numerical reduction in risk of progression was 20% in patients after last antiestrogen therapy when combined with metformin (HR=0.8, 95% CI 0.2 to 2.7; Table 3, Figure 4). No significant difference was observed, with a limited number of patients in each subgroup.

**Table 3:** Subgroup analysis

		N	PFS	HR	95%CI	P
**Age**						
<60 year	AI+Met	16	6.5	1.09	0.5-2.2	0.810
	AI	21	6.9			
≥60 year	AI+Met	14	3.2	1.49	0.6-3.7	0.391
	AI	9	5.2			
**No. of metastatic sites**						
=1	AI+Met	12	6.5	0.62	0.2-1.5	0.327
	AI	11	5.8			
≥2	AI+Met	18	2.8	1.58	0.7-3.3	0.218
	AI	19	6.9			
**Previous chemotherapy for MBC**						
≤1	AI+Met	24	6.5	0.95	0.6-2.5	0.758
	AI	29	6.2			
≥2	AI+Met	6	2.0	1.68	0.2-16.1	0.649
	AI	1	2.3			
**Purpose of most recent therapy**						
Adjuvant therapy	AI+Met	14	7.8	0.97	0.4-2.5	0.067
	AI	8	5.2			
Treatment of MBC	AI+Met	16	2.4	1.62	0.8-3.4	0.211
	AI	22	6.2			
Resistance to endocrine therapy^**^						
Primary resistance	AI+Met	3	10.8	0.71	0.1-4.4	0.714
	AI	5	5.8			
Secondary resistance	AI+Met	24	3.2	1.21	0.6-2.1	0.719
	AI	21	5.3			
**Most recent therapy**						
Antiestrongen	AI+Met	5	10.8	0.80	0.2-2.7	0.722
	AI	10	4.3			
AI	AI+Met	25	2.8	1.38	0.7-2.6	0.320
	AI	20	6.2			
**Overall**	AI+Met	30	4.7	1.21	0.7-2.1	0.487
	AI	30	6.0			

^**^ Primary endocrine resistance is defined as: a relapse while on the first 2 years of adjuvant endocrine therapy, or PD within first 6 months of first-line endocrine therapy for MBC, while on endocrine therapy. Secondary (acquired) endocrine resistance is defined as: a relapse while on adjuvant endocrine therapy but after the first 2 years, or a relapse within 12 months of completing adjuvant endocrine therapy, or PD ≥6 months after initiating endocrine therapy for MBC, while on endocrine therapy.

### Safety

Median drug exposure times were 3.8 months (range, 1.5 to 32.0 months) in the combination group and 6.0 months (range, 2 to 30.7 months) in the control group. Both combination and monotherapy were well tolerated. No substantial differences in the incidence and severity of adverse events (AEs) were obtained between the two treatment groups (Table 4). The most common AEs were arthralgia (n=5; 16.7%) and headache (n=3; 10.0%) in the combination group, and arthralgia (n=3; 10.0%) in the control group. Grade 3/4 AEs were anemia (n=1; 3.3%) and thrombocytopenia (n=1; 3.3%) in the combination group. No treatment related serious AEs were observed in this study. One patient in the combination group discontinued the treatment for grade 3 anemia and grade 4 thrombocytopenia, which was considered to be related to disease progression. This patient received symptomatic treatment, including blood transfusion, but died of myelosuppression.

**Table 4:** Adverse event

AE	AI+Met				AI			
	Grade 1	Grade 2	Grade 3	Grade 4	Grade 1	Grade 2	Grade 3	Grade 4
Anemia	0	0	1(3.33%)	0	0	0	0	0
Neutropenia	0	0	0	0	1(3.33%)	0	0	0
Thrombocytopenia	0	0	0	1(3.33%)	0	0	0	0
Fever	1(3.33%)	0	0	0	1(3.33%)	0	0	0
Diarrhea	1(3.33%)	1(3.33%)	0	0	0	0	0	0
Anorexia	1(3.33%)	0	0	0	1(3.33%)	0	0	0
Fatigue	1(3.33%)	0	0	0	2(6.67%)	0	0	0
Nausea and vomiting	2(6.67%)	0	0	0	0	0	0	0
Abdominal distension	1(3.33%)	0	0	0	0	0	0	0
Constipation	1(3.33%)	0	0	0	0	0	0	0
Arthralgia	5(16.7%)	0	0	0	3(10.0%)	0	0	0
Alopecia	0	0	0	0	0	0	0	0
Rash	0	0	0	0	1(3.33%)	0	0	0
Edema	0	0	0	0	1(3.33%)	0	0	0
Liver enzyme escalation	2(6.67%)	0	0	0	2(6.67%)	0	0	0
Myalgia	0	0	0	0	1(3.33%)	0	0	0
Headache	3(10.0%)	0	0	0	0	0	0	0
Skin Pruritus	0	0	0	0	1(3.33%)	0	0	0
Cough	0	1(3.33%)	0	0	0	0	0	0
Insomnia	1(3.33%)	0	0	0	0	0	0	0
Hand-foot syndrome	0	1(3.33%)	0	0	0	0	0	0

## DISCUSSION

This phase II randomized clinical trial failed to show an improved efficacy after metformin addition. PFS was 4.7 months in the AI and metformin group, and 6.0 months in the control group, with no significant difference. In subgroup analysis, no favorable PFS for the combination group was observed. Similarly, no significant increase was found in ORR, CBR and OS. As expected, the incidence rates of AEs were identical in two groups.

Few studies have confirmed efficacy outcome data for metformin in breast cancer patients. Only two studies indicated that metformin improves pCR rate when combined with chemotherapy in the neoadjuvant setting. A retrospective study showed that diabetic patients with concurrent metformin in neoadjuvant chemotherapy have a higher pCR rate than diabetic and non-diabetic patients not administered metformin (24% vs. 8% and 16%, respectively, p = 0.02) [14]. Another Chinese phase II randomized clinical trial showed increased pCR after metformin addition (20% vs 6.7%, p=0.0119) [15].

However, most previous studies focused on surrogate biomarkers of cell proliferation, cell apoptosis, or AMPK/mTOR signaling affected by metformin as neoadjuvant therapy. The majority of these studies showed reduced Ki67 levels, increased TUNEL staining [17], increased mean AMPK scores, and decreased pAKT scored [16]. Nevertheless, other trials reported conflicting outcomes. A phase II RCT of 200 patients found no significant difference in Ki67 comparing metformin treatment with controls. Interestingly, metformin significantly decreases ki-67 in women with HOMA (homeostatic model assessment, to evaluate status of insulin resistance) > 2.8 or high body mass index (BMI), indicating that metformin's effects may differ according to insulin resistance and the metabolic characteristics of patients [21]. The above translational studies mainly indicated that metformin exerts antitumor activity by inhibiting cell proliferation and promoting apoptosis, although further studies are required for confirmation. To this end, numerous clinical trials are ongoing, involving metformin as monotherapy (NCT01101438, NCT01905046), or combined with anti-tumor agents such as docetaxel, epirubicin and cyclophosphamide (NCT01929811), letrozole (NCT01589367), ganitumab (NCT01042379), and sirolimus (NCT02145559).

Though all pre-specified subgroups showed no significant differences, a numerical PFS increase in the combination group was observed in patients with primary resistance and those progressing on last antiestrogen therapy. Patients with primary resistance seemed to be more responsive to metformin, although there was a limited number of patients in this subgroup. Previous studies indicate that metformin inhibits the proliferation of endocrine-resistant breast cancer cell lines, and these effects may be more pronounced in patients with primary resistance. First, metformin could induce apoptosis and inhibit proliferation mediated by AMPK signaling in endocrine therapy resistant HR-positive breast cancer cell lines [22]. Secondly, the mechanisms of endocrine resistance differ between primary and secondary resistance. Primary resistance mainly includes lack of ERα expression, while secondary resistance is associated with a plethora of mechanisms, and increased crosstalk receptor tyrosine kinase signaling (EGFR, ERBB2, IGF1R and PI3K pathways) may play an important role [23, 24]. Most importantly, with more crosstalk proliferation pathways involved in secondary resistance, growth inhibition and apoptosis induction by metformin may be weakened, or even eliminated. Breast cancer with secondary resistance often shows induced IGF1R signaling [25], which may also explain metformin resistance [26]. Activation of the IGFR-1/IRS-1 axis results in elevated cell survival signals, thus counteracting the antitumor activity of metformin [26].

Patients progressing on last antiestrogen therapy had somewhat prolonged PFS in the combination group. This was consistent with subgroup analysis data in BOLERO-2. Previous studies suggested that mTOR inhibitors may have favorable effects in antiestrogen resistant breast cancer compared with the AI resistant counterpart. A pre-clinical study compared the growth inhibition effects of everolimus in TAM-R (tamoxifen resistance model) and MCF7-X (estrogen deprivation resistance model), and found higher inhibition rates and lower half maximal inhibitory concentrations in the TAM-R group compared with the MCF7-X model. Besides its inhibition of mTOR, metformin decreases basal and insulin-stimulated aromatase expression, which is an important mechanism of its usefulness in polycystic ovary syndrome [27]. Metformin may synergistically act with aromatase inhibitors by further inhibiting aromatase. Changing another mechanism of endocrine therapy, from antiestrogen to aromatase inhibition, had higher antitumor effects in patients who had prior antiestrogen therapy compared with those who had prior steroidal AI. These effects were enhanced by metformin. However, the above interpretations are mostly based on preliminary studies, and the number of patients in every subgroup is too small to detect differences. Therefore, additional clinical studies are required to confirm these findings.

The current study showed a negative outcome, and was incapable to confirm an enhanced anti-tumor activity after metformin addition. This could be attributed to the following reasons. On the one hand, we administered metformin at the conventional dose as used in type 2 diabetes, since 0.5g bid PO proves to be a safe dose after decades of clinical use among type 2 diabetes. Previous studies showed that much higher concentrations of metformin are needed in order to exert its direct effects on AMPK–mTOR [28–31]. Results from two xenograft models reported that the human equivalent of 1500–2250 mg/day is needed to inhibit tumorigenesis [32–34]. The above studies administered metformin in the neoadjuvant setting at doses between 1500 mg/d-2000 mg/d as a monotherapy or in combination [14, 16, 21, 35, 36]. But pitifully, there were not any clinical study we could refer to when we designed this study in 2011. Therefore, we chose a safe dose of metformin in this exploratory study. The dose in this study seems insufficient to yield full anti-tumor activity and reverse resistance to endocrine therapy. Actually, in our Center, further dose-escalation study to confirm optimal dose of metformin combined with aromatase inhibitors among ER+HER2- advanced breast cancer patients is ongoing.

One the other hand, indirect, insulin-dependent effects may play a more important role in anticancer effects of metformin compared with direct, insulin-independent effects. In addition, the majority of studies indicated that metformin is most effective in patients with high BMI and insulin resistance [21]. In the current study, the BMIs of patients were relatively normal (median 22.1, range 18.7-26.5) and few had metabolic disorders like diabetes. Therefore, the benefits from insulin-dependent effects may be limited in the current patient population. The main limitation of this study is that the HOMA index and other metabolic markers were not assessed, making it difficult to accurately identify which subtype of patients can benefit from such treatment. Moreover, overactivation of the IGF1R pathway is one of the key mechanisms of acquired resistance among HR-positive patients [25]. Enhanced IGF1R activity promotes cell survival and weakens the antitumor activity of metformin [26].

This study was a pioneer work, providing preliminary data of the effects of metformin plus AI in postmenopausal patients with hormone receptor positive metastatic breast cancer. Overall, the results suggested that metformin does not enhance efficacy in terms of PFS, ORR, CBR and OS, and does not increase the incidence rates of adverse events, when combined with AI. Patients with primary resistance and progressing on last antiestrogen therapy showed a trend of prolonged PFS. However, these data should be interpreted in the context of limited statistical power provided by a relatively small sample size. Further studies with sufficient metformin amounts, measurements of metabolic markers, appropriate populations, and larger sample sizes are warranted in the future.

## MATERIALS AND METHODS

### Study design and treatments

This was a prospective, open-label, randomized, single-center, phase II trial comparing aromatase inhibitors plus metformin and aromatase inhibitors plus placebo in HR-positive postmenopausal patients with metastatic breast cancer pretreated with endocrine therapy. Eligible patients were randomly assigned 1:1 to one of the following 2 treatment arms: aromatase inhibitor (letrozole 2.5 mg/d orally or exemestane 25 mg/d orally) plus metformin (0.5g bid, orally) or aromatase inhibitor plus placebo. The dose of metformin administered reflected the conventional dosage used in type 2 diabetes. Letrozole was administered to patients experiencing progression on/after tamoxifen or steroid aromatase inhibitor (SAI) treatment. Exemestane was administered to patients with previous non-steroid aromatase inhibitor (NSAI) treatment. Four weeks were defined as a treatment cycle. The patients received treatment until disease progression, intolerable toxicity, or voluntary refusal. The current study was performed in accordance with the Declaration of Helsinki and consistent with the International Conference on Harmonization of Technical Requirements for Registration of Pharmaceuticals for Human Use (ICH) Good Clinical Practice. The study protocol, patient consent forms, and information sheets were approved by the relevant independent ethics committees and institutional review boards. All patients provided written informed consent before enrolment (ClinicalTrials.gov identifier: NCT01654185).

### Patients

Eligible patients were postmenopausal women with locally advanced or metastatic, pathologically confirmed ER and/or PgR positive breast cancer. Patients who experienced progression after prior endocrine therapy in adjuvant or metastatic settings were allowed to be enrolled. Measurable lesions were not necessary in eligible patients. Patients treated with chemotherapy for advanced disease were also allowed. In addition, all eligible patients were required to have an ECOG score of 0 to 1, evidence of adequate organ function, and life expectancy of at least 3 months.

Exclusion criteria were: HER2 overexpression (3+ status by immunohistochemistry or amplification ratio ≥2.0 by fluorescence *in situ* hybridization); disease progression on chemotherapy for advanced breast cancer, or less than 2 week washout period for the last endocrine therapy after chemotherapy; life-threatening visceral metastases or central nervous system metastases; radiotherapy within 4 weeks of randomization (palliative radiotherapy for bone metastasis within 2 weeks was permitted); current or prior malignancy (except breast cancer, adequately treated skin cancer, or *in situ* carcinoma of the cervix); treatment with other experimental drugs before randomization; long-term systemic steroid therapy; prior or present metformin use for blood glucose control; age ≥70 with renal hypofunction or any severe concomitant conditions.

### Efficacy and tolerability

The primary endpoint was progression free survival (PFS), defined as the time from randomization to objective disease progression or death for any cause before documented disease progression. Secondary end points were objective response rate (ORR, proportion of patients with complete or partial response), clinical benefit rate (CBR, proportion of all patients with complete response, partial response, or stable disease for at least 24 weeks), overall survival (OS, time interval from random assignment to death in follow-up), and tolerability.

Tumor assessment was performed by computed tomography, spiral computed tomography, or magnetic resonance imaging, at baseline and every two cycles until disease progression or death occurred, according to RECIST 1.1. Safety and tolerability were assessed at each cycle; incidence and frequency of adverse events (AEs) were recorded throughout the study. AEs were graded using the National Cancer Institute Common Terminology Criteria for Adverse Events (version 4.0).

### Statistical analysis

Sample size calculation was based on the primary endpoint of PFS, assuming exponential progression times. To detect a prolongation of 1.38 months in median PFS for AI plus metformin over AI, at a two-sided significance level of 5% with 80% power, at least 30 patients in each arm were required within 12 months, with 10% dropout rate.

For the primary endpoint of PFS, Kaplan-Meier plots revealed median PFS estimates for each treatment group. The primary analysis was an unadjusted log-rank test. The treatment effect was estimated using COX proportional hazards model and expressed as hazard ratio of AI plus metformin versus AI plus placebo. Subgroup analysis used the Cox proportional hazards model, stratified by the following pre-defined covariates: age (<60y vs. ≥60y), number of metastatic sites (1 vs. ≥2), lines of previous chemotherapy (≤1 vs. ≥2), purpose of the most recent therapy (adjuvant therapy vs. treatment of MBC), resistance to endocrine therapy (primary vs. secondary resistance), and last endocrine therapy before the study treatment (antiestrogen vs. aromatase inhibitor). According to the ESO-ESMO 3^rd^ international consensus guidelines for advanced breast cancer (ABC3), primary endocrine resistance is defined as a relapse in the first 2 years of adjuvant endocrine therapy, or PD within the initial 6 months of first-line endocrine therapy for MBC, while on endocrine therapy. Secondary (acquired) endocrine resistance is defined as a relapse while on adjuvant endocrine therapy but after the first 2 years or within 12 months of completing adjuvant endocrine therapy, or PD ≥6 months after initiating endocrine therapy for MBC, while on endocrine therapy. Efficacy analyses were performed according to the intention-to-treat principle, with all randomized patients included for the primary endpoint.

ORR and CBR were assessed by the logistic regression model. Results were expressed as odds ratio of AI plus metformin versus AI plus placebo, with the corresponding 95% CI and P value. OS was evaluated by unadjusted log-rank test as described for PFS analysis. The log-rank test was performed when approximately 50% of the patients had died, which occurred at the time of the present PFS analysis. The two study arms were compared in incidence rates of certain pre-specified categories of adverse events by two-sided Fisher's exact test at nominal significance of P =0.05.

## Abbreviations

HR: hazard ratio
CI: confidence interval
CR: complete response
PR: partial response
SD: stable disease
PD: progression disease
PFS: progression-free survival
CBR: clinical benefit rate
OR: odds ratio
ORR: objective response rate
OS: overall survival
mTOR: mammalian target of rapamycin
AMPK: AMP-activated protein kinase
pCR: pathologic complete response
TUNEL: terminal deoxynucleotidyl transferase dUTP nick end labeling
PI3K: phosphatidylinositol 3-kinase
CFDA: China Food and Drug Administration
HR-positive: hormone receptor (HR) positive
AI: aromatase inhibitor
AE: adverse event
HOMA: homeostatic model assessment
BMI: body mass index
